# Complex pattern of genetic structuring in the Atlantic salmon (*Salmo salar* L.) of the River Foyle system in northwest Ireland: disentangling the evolutionary signal from population stochasticity

**DOI:** 10.1002/ece3.32

**Published:** 2011-11

**Authors:** Dennis Ensing, Paulo A Prodöhl, Philip McGinnity, Patrick Boylan, Niall O'Maoiléidigh, Walter W Crozier

**Affiliations:** 1 Agri-Food and Biosciences Institute Northern Ireland, Fisheries and Aquatic Ecosystems BranchNewforge Lane, Belfast, Northern Ireland, BT9 5PX; 2 School of Biological Sciences, Queen's UniversityBelfast, Northern Ireland, BT9 7BL; 3 Department of Zoology, Ecology & Plant Science/Aquaculture & Fisheries Development Centre, University CollegeCork, Ireland; 4 Loughs AgencyLondonderry, Northern Ireland, BT47 2AB; 5 Marine InstituteDublin 2, Ireland

**Keywords:** Anthropogenically induced genetic changes, *F*_ST_ and *D*_est_, Isolation-by-Distance, Metapopulation and member-vagrant models, *Salmo salar*, Within-river population structure

## Abstract

Little is known about the microevolutionary processes shaping within river population genetic structure of aquatic organisms characterized by high levels of homing and spawning site fidelity. Using a microsatellite panel, we observed complex and highly significant levels of intrariver population genetic substructure and Isolation-by-Distance, in the Atlantic salmon stock of a large river system. Two evolutionary models have been considered explaining mechanisms promoting genetic substructuring in Atlantic salmon, the member-vagrant and metapopulation models. We show that both models can be simultaneously used to explain patterns and levels of population structuring within the Foyle system. We show that anthropogenic factors have had a large influence on contemporary population structure observed. In an analytical development, we found that the frequently used estimator of genetic differentiation, *F*_ST_, routinely underestimated genetic differentiation by a factor three to four compared to the equivalent statistic Jost's *D*_est_ (Jost 2008). These statistics also showed a near-perfect correlation. Despite ongoing discussions regarding the usefulness of “adjusted”*F*_ST_ statistics, we argue that these could be useful to identify and quantify qualitative differences between populations, which are important from management and conservation perspectives as an indicator of existence of biologically significant variation among tributary populations or a warning of critical environmental damage.

## Introduction

Over the past decade, microsatellites have been used widely as the molecular marker of choice for studies of population structure in Atlantic salmon (*Salmo salar*). The spatial scales of these studies have varied from intercontinental (King et al. 2001), intracontinental (Spindle et al. 2003), to regional (e.g., Fontaine et al. 1997; Dillane et al. 2007). Given their greater discriminatory power in comparison to other molecular markers (e.g., protein electrophoresis, Heggberget et al. 1986), microsatellites are particularly effective for studies examining population genetic structure on small spatial scales (e.g., Wright and Bentzen 1995; O'Connell and Wright 1997). A small number of studies (Garant et al. 2000; Primmer et al. 2006; Vähä et al. 2007; Dillane et al. 2008) have focused on within-river population structure in Atlantic salmon, and these differed considerably, for instance, in the number of microsatellite markers used, total number of samples, temporal replicate sampling, river catchment size, geomorphological structure, and life-history stage targeted (Table 1).

**Table 1:** Comparison of sampling design and geography of studied area in recent studies examining evolutionary models explaining within-river population structure in Atlantic salmon (*Salmo salar*)

	Garant et al. (2000)	Primmer et al. (2006)	Vaha et al. (2007)	Dillane et al. (2008)	This study
System studied	Sainte-Marguerite	Varzuga	Teno/Tana	Moy	Foyle
Number of loci	5	17	32	12	7
Number of samples	683	392	792	1606	1086
Geomorphological feature	Linear system	Linear system	Linear system	Lacustrine system	Dendritic system
Catchment size	1114 km^2^	9510 km^2^	16,386 km^2^	2000 km^2^	4450 km^2^
Life stage sampled	Fry	Parr	Adult	Fry/Parr	Fry/Parr
Temporal genetic stability test	Yes	No	No	Yes	Yes
Evolutionary model	Meta population/Member Vagrant	Member Vagrant	Metapopulation	Metapopulation	Metapopulation/Member Vagrant
Climatic zone	Subarctic	Arctic/Subarctic	Arctic/Subarctic	Cool temperate	Cool temperate
Anthropogenic disturbance	Moderate (hydro power)	Limited	Limited	Substantial (drainage)	Substantial (drainage)

A common finding of these studies, to a greater or lesser degree, was the existence of within river genetic population structure. For instance, in the west of Ireland, within-river genetic variation has been reported in the River Moy. Dillane et al. (2008) observed a considerable degree of genetic variation within and between tributaries of this comparatively large and complex river system, with the presence of two large lakes as a significant landscape feature, explaining the population structure observed. Over the past few years, a number of similar studies have attempted to identify the best underlying evolutionary models responsible for observed patterns of genetic structuring of wild Atlantic salmon populations (Garant et al. 2000; Primmer et al. 2006). Two evolutionary models have often been proposed to describe the underlying cause of such structuring; the “metapopulation” and the “member/vagrant evolutionary models.” Under the metapopulation model, local subpopulations are linked by gene flow and experience recurring extinction–recolonization events due to environmental instability (Hanski 1998). Consequently, strong genetic structuring and locally adapted gene pools are prevented from being formed. A temporally stable genetic population structure and a positive correlation between genetic differentiation and geographical distance (Isolation-by-Distance, IBD) are unlikely to occur in a true metapopulation.

The member-vagrant evolutionary model predicts that precise homing restricts gene flow, thus favoring the formation of locally adapted gene pools and subsequent strong and temporally stable genetic structuring among populations, with a clear IBD signal (e.g., Sinclair 1988; Garant et al. 2000). Primmer et al. (2006) reported on strong, well-supported population structuring in combination with clear IBD in the Atlantic salmon population of the Varzuga River (Russia) and concluded that this was in accordance with the predictions of the member-vagrant evolutionary model though, one of the specific predictions of the member-vagrant model, namely temporal stability, was not tested by Primmer et al. (2006). Garant et al. (2000) reported weak genetic structuring and lack of IBD in the Canadian Sainte-Marguerite River, and found evidence for temporal stable genetic substructuring at four of seven sampling locations, concluding that both the member-vagrant and the metapopulation models can be used to describe the genetic structure observed.

Knowledge of the evolutionary model most suitable to explain observed genetic differentiation in salmonid populations can be of great benefit in designing and implementing effective management and conservation strategies to ensure long-term sustainability of fish stocks (e.g., to identify and assess the biological and genetic health status of individual population units). Given the lack of consensus as to which evolutionary model best explains patterns of genetic differentiation in wild Atlantic salmon populations, and that studies focusing on within-river genetic structure in this species remain relatively scarce, more studies are warranted.

The River Foyle system, in the northwest of Ireland, was, until recently, one of the most productive wild Atlantic salmon fisheries in Western Europe. Annual commercial catches of salmon in the estuary and the adjacent sea area have ranged between 12,176 and 44,000 salmon between 1997 and 2006 (Loughs Agency Annual Report and Accounts 2007), while annual angling catches for the River Foyle were estimated to be in the region of 10,000 fish. There has been a cessation of commercial salmon fishing, seaward of Lough Foyle since 2007 in order to reduce the potential for the exploitation of mixed stocks outside of the Foyle, thus changing the focus of exploitation toward a single river fishery with an identifiable surplus. As a result, commercial catch figures dropped to 12,176 fish in 2006 and 5372 in 2007 (Loughs Agency Annual Report and Accounts 2007). From 2010, commercial fishing on the River Foyle has been completely banned as a conservation measure.

The Foyle system, in contrast to the Moy, Varzuga, Teno, and Sainte-Marguerite (Table 1), is a temperate (geographically), medium-sized, geomorphologically complex system, comprising an extensive dendritic network of major and minor tributaries rivers with only one significant lake (Lough Derg), which is a headwater lake upstream of available salmon spawning habitat and thus unlikely to contribute to population structure, representing a variety of habitat types and flow regimes. Returning adult spawners in the River Foyle system consist mainly of grilse (one-sea winter; 1SW) with one of the larger tributaries (River Finn) having a major component of spring-running multi-sea-winter (MSW) salmon. Stocks in several of the tributaries display characteristically late running grilse components. Thus, this complex river system provides an excellent opportunity to examine further evidence for the influence of microevolutionary processes shaping contemporary population genetic structure, which both contrast and complement previous studies.

The specific aims of this study are to: (1) determine spatial genetic structure in the Foyle system, (2) asses which evolutionary model (member-vagrant or metapopulation) best fits the observed results, and (3) compare and discuss results with other such studies from the recent literature.

## Materials and Methods

### Study site

**Figure 1:**
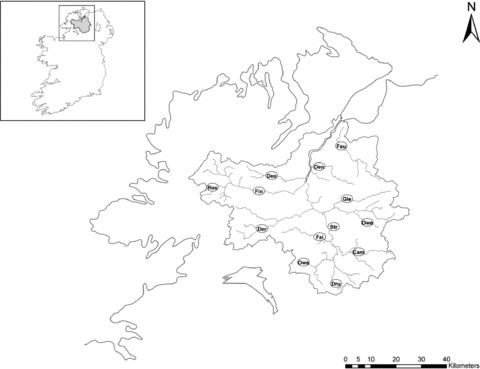
Map of Ireland (inset) and the Foyle River system. Symbols indicate location of sampling sites. Abbreviations are as given in Table 2.

The Foyle River system (55°00′N; 07°20′W) is located in the northwestern corner of the island of Ireland, forming part of the land border between Northern Ireland (United Kingdom) and the Republic of Ireland (Fig. 1). The Foyle River system, together with the Faughan River, falls within a single management jurisdiction, the cross-border Loughs Agency. The river has a catchment area of 4450 km^2^. The main tributaries of the Foyle are the rivers Finn and Derg to the west, and the Owenkillew River in the eastern part of the catchment. The Foyle main stem is known under different names throughout its course. Downstream of the confluence of the Drumragh River and the Camowen River, the main stem is called River Strule until it meets the River Derg downstream of which the main stem is known as River Mourne. After the River Finn joins the River Mourne, the main stem is called River Foyle until it drains into Lough Foyle. The Faughan River does not join the Foyle main stem, but directly enters Lough Foyle to the east of the main river.

### Sampling strategy

Sampling was conducted throughout the Foyle system, including the adjacent River Faughan, with an average waterway distance between the sampling locations of 72 ± 28 (SD) km and a range of 17.8–132.4 km. Following consultation with local fishery managers, sample coverage included most known spawning and nursery areas in all major tributaries. Sampling by electrofishing conducted during 2006 and 2007 captured juvenile Atlantic salmon from multiple year classes; 48 or 96 fish per sampling location with ideally a 50/50 ratio of 0^+^ (fry) and 1^+^ (parr) fish. A total of 13 sampling sites were visited. Among these, two temporal replicate samples originally taken at two sampling sites (Deele and Faughan Rivers) in 1999 were included to test for temporal variation. In total, 1086 individual tissue samples were collected for this study (Fig. 1; Table 2). The idealized sampling ratio was not achieved at every sampling location, indeed at some sampling sites visited during 2007 no 0^+^ sample was taken due to the fact that at the time of year of the visit, the 0^+^ Atlantic salmon could not yet be distinguished from 0^+^ brown trout (*S. trutta*). The aim was to collect 96 samples per sampling location. At some of the (smaller) tributaries, this number was not reached due to smaller numbers of juvenile salmon present. In these cases, the number of samples taken was reduced to 48.

**Table 2:** Details of sampling sites in the Foyle River system

Site	Abbreviation	Sample year	Coordinates	Region	*N*
Faughan	Fau	2006	54°57′N; 07°17′W	Northeast	96
Faughan	Fau_99	1999	54°57′N; 07°17′W	Northeast	45
Deele	Dee	2007	54°51′N; 07°41′W	Northeast	48
Deele	Dee_99	1999	54°51′N; 07°41′W	Northeast	33
Burn Dennet	Den	2006	54°53′N; 07°23′W	Northeast	96
Strule (Cappagh Burn)	Str	2007	54°40′N; 07°18′W	Northeast	48
Owenreagh East	Owe	2006	54°41′N; 07°05′W	Northeast	48
Camowen	Cam	2007	54°34′N; 07°09′W	Northeast	48
Finn	Fin	2006	54°47′N; 07°45′W	West	96
Reelan	Ree	2006	54°49′N; 08°03′W	West	96
Fairy Water	Fai	2007	54°38′N; 07°23′W	West	48
Derg	Der	2006	54°39′N; 07°44′W	West	96
Owenreagh South	Ows	2006	54°32′N; 07°29′W	Southeast	96
Glenelly	Gle	2006	54°46′N; 07°13′W	Southeast	96
Drumragh	Dru	2007	54°28′N; 07°17′W	Southeast	96

The sampling of multiple year-classes combined with taking the samples from river stretches of several hundred meters minimized the likelihood for the “Allendorf-Phelps effect” (Allendorf and Phelps 1981), whereby false statistically significant genetic differentiation among sampling sites can result from the sampling of the offspring of a very limited number of individuals that do not represent the full extent of genetic variation present in the population.

Tissue samples from the juvenile Atlantic salmon were taken by removing the adipose fin before releasing the fish in the case of the parr samples, or by sacrificing the whole fish for the fry samples. In both cases, the tissue samples were preserved in molecular grade 99% ethanol until analysis.

### Genetic analysis

DNA was extracted by using a Chelex100 (Bio-Rad, Hemel Hempstead, United Kingdom) protocol (Estoup et al. 1996) on small amounts (∼3 mm^2^) of fin tissue. Extracted DNA was kept frozen at –20°C until amplification using a polymerase chain reaction (PCR). Samples were screened for a suite of seven highly polymorphic microsatellite loci; Sssp1605, Sssp2201, Sssp2210, Sssp2216, SsspG7 (Paterson et al. 2004), Ssa197, and Ssa202 (O'Reilly et al. 1996). The forward primer of each primer pair was labeled at the 5′ end with a fluorescent dye: 6FAM (blue) for Ssa197, Sssp1605, Sssp2201, and Sssp2210 loci, VIC (green) for loci Sssp2216 and SsspG7, NED (yellow) for locus Ssa202.

The individual PCR amplification reactions for loci SsspG7, Ssa202, and Ssa197 consisted of 1 µl of extracted template DNA, 0.2 mM dNTP's (Invitrogen, Paisley, Scotland, United Kingdom), 0.7× reaction buffer (13 mM Tris-HCl [pH 8.4], 33 mM KCl; Invitrogen), 1.5 mM MgCl_2_, 0.3 µM forward and reverse primer (0.15 µM in the case of Ssa197), 0.13 U *Taq* DNA polymerase (Invitrogen), and ultraPURE H_2_O (Gibco, Paisley, Scotland, United Kingdom) to a total reaction volume of 15 µl. For the remaining four loci, the PCR reaction consisted of 1 µl of extracted template DNA, 0.2 mM dNTP's (Invitrogen), 1× reaction buffer (20 mM Tris-HCl [pH 8.4], 50 mM KCl; Invitrogen), 5 mM MgCl_2_, 0.38 µM of the forward and reverse primer for Sssp1605, 0.95 µM for Sssp2201, 0.17 µM for Sssp2210, 0.88 µM for Sssp2216, 0.1 U of *Taq* DNA polymerase, and ultraPURE H_2_O to a total reaction volume of 10 µl. The PCR, in which Sssp2210/Sssp2201 and SsspG7/Ssa197 were multiplexed, was performed using the following amplification cycles: denaturing at 95°C/2 min followed by 33 cycles of 95°C/45 sec, 58°C/45 sec, 72°C/45 sec, which was followed by a final cycle of 95°C/45 sec, 58°C/45 sec, 72°C/5 min on a Thermo Hybaid PCR Express thermocycler (Thermo Hybaid, Franklin, MA, United States).

The PCR products were prepared for electrophoresis on an ABI 3130 Automatic DNA Analyzer (Applied Biosystems, Paisley, Scotland, United Kingdom) according to the manufacturer's instructions using ABI GS-500 LIZ internal size standard. Alleles were scored and sized using the automated bin function of the ABI GeneMapper v4.0 software, but were manually checked and verified.

### Quality control

Microchecker v2.2.3 (van Oosterhout et al. 2004) was used to test for the possible presence of null-alleles and allele scoring errors. To further check for genotyping errors, 118 individuals (11% of the total data) from two randomly chosen sampling locations were genotyped again following the same protocols as in the original analysis. The genotypes were compared to the previous genotypes and allelic mismatches were counted to establish an error rate for the genotyping in this study.

### Statistical analysis

Genetic diversity in all samples was calculated; number of alleles (*N*_A_), allelic frequencies, observed (*H*_O_), and expected (*H*_E_) heterozygosity, using the program GENEPOP v3.4 (Raymond and Rousset 1995). Deviations from Hardy–Weinberg equilibrium (HWE) for each sample (Guo and Thompson 1992) were calculated using the Genepop 3.4 software (Raymond and Rousset 1995).

Temporal genetic stability, between cohorts (i.e., short-term temporal stability) and between samples Deele and Faughan and their 1999 equivalents, was assessed by testing for pairwise heterogeneity in allelic frequency distributions between temporal samples with GENEPOP v3.4. The same program was also used to test for genotypic linkage disequilibrium across putative populations, to confirm independent allelic segregation among different marker loci.

Genetic differentiation between samples was quantified by calculating pairwise *F*_ST_ values, or variance in allele frequencies (Weir and Cockerham 1984), using FSTAT v2.9.3. (Goudet 2001). The statistical significance of estimated values was tested by permutation. The same programme, FSTAT v2.9.3., was also used to calculate allelic richness (*A*_R_), which is the allele number per population corrected for differences in sample size. Corrections to account for multiple tests, sequential Bonferroni correction, (Rice 1989) were carried out for all tests mentioned above.

Genetic differentiation between samples was also calculated using an unbiased estimator of Jost's *D* (Jost 2008) as implemented in the programme SMOGD (Crawford 2010); *D*_est_. Recently, the validity of using *F*_ST_ and related measures of genetic differentiation in population genetic studies based on microsatellite data has been questioned (e.g., Hedrick 2005; Jost 2008; Whitlock 2011). The rationale is that *F*_ST_ tends to be biased toward lower values, particularly in situations where within-locus genetic diversity is high, as is often the case for highly polymorphic microsatellite data (see Hedrick 2005 and Jost 2008). Here, we compare *D*_est_ directly to *F*_ST_ to assess whether “traditional” measures of population differentiation underestimate differentiation in the current dataset, to what degree, and what possible consequences this has for further analysis and conclusions. For this purpose, possible correlations between *F*_ST_ and *D*_est_ were compared by means of a Mantel test (Mantel 1967).

Genetic distances between samples were estimated using the Cavalli-Sforza and Edwards (1997) Chord distance measure (*D*_C_) as implemented in the programme POPULATIONS v1.2.30beta (Langella 1999). This measure of genetic distance was selected in favor of others as *D*_C_ is drift based, relatively independent from a specific mode of mutation of alleles and fluctuations in effective population size, and is most likely to provide the correct phylogeny in closely related populations (e.g., Takezaki and Nei 1996; Goldstein and Pollock 1997). The distance matrix was used to construct a neighbor-joining (NJ) phylogram with 10,000 bootstrap replicates over loci to obtain confidence estimates of the branching pattern, drawn with the programme TREEVIEW (Page 1996). To test for genetic IBD, pairwise linearized *F*_ST_ and *D*_est_ estimates, calculated between sample sites, were plotted against geographical distance using a Mantel test (Mantel 1967) implemented in IBDWS v3.15 (Jensen et al. 2005). The significance of observed associations were evaluated using 10,000 permutations.

To further investigate the level of population genetic structuring within the River Foyle system, the programme STRUCTURE v2.3.1 (Pritchard et al. 2000) was also used. The algorithm built within this programme allows for the determination of the most likely number of populations (clusters) explaining the data without requiring “a priori” definition of populations. The programme was set to run using the admixture model with correlated gene frequencies. As STRUCTURE uncovers only the uppermost level of structure in a dataset, Pritchard and Wen (2004) suggest running STRUCTURE subsequently on each identified cluster separately to reveal any underlying structure; in effect a “hierarchical structure analysis.” This approach was followed in this study.

To infer the true number of clusters (*K*), we followed Evanno et al's. (2005)“ad-hoc” statistic (Δ*K*) based on the rate of change in the log probability between consecutive *K* values, which ranged from *K* = 1 to *K* = 20 with a burn-in and Markov chain Monte Carlo length of 10,000 each (100,000 burn-in and 200,000 Markov chain Monte Carlo replicates when analyzing for hierarchical within-cluster structure) for 20 runs per *K*-value. The modal value of the Δ*K* distribution is assumed to represent the true number of clusters or populations in the data. The reliability of the replicate analysis mentioned above was enhanced by use of the CLUMPP 1.1.2 (Jakobsson and Rosenberg 2007) software package. This computer program's “Greedy” algorithm was used to find the optimal alignment of the results from the 20 replicate cluster analyses. A second method used to find the most likely number of clusters was taking the maximum value of the mean value of the log likelihood (L[*K*)] of the data as the most likely value of *K* (e.g., Zeisset and Beebee 2001; Dillane et al. 2008).

The results from the STRUCTURE analysis were summarized using the programme Distruct 1.1 (Rosenberg 2004), which generates a bar plot of the STRUCTURE output data where each individual is represented by vertical colored line. Different colors code for each different *K* representing the individual's membership coefficients to each *K*. A hierarchical analysis of spatial genetic diversity was performed using the method of molecular variance (AMOVA) implemented with the software Arlequin 3.1.1 (Excoffier et al. 2005).

Barriers to gene flow that shape patterns of genetic differentiation between populations were identified using the software package Barrier 2.2 (Manni et al. 2004), which utilizes Monmonier's maximum difference algorithm to identify such barriers. The robustness of the computed barriers was assessed by analyzing 1000 resampled bootstrapped *F*_ST_ matrices with the software package.

## Results

### Quality control

No evidence for either large allele dropout or scoring errors due to stuttering was detected by MicroChecker. In the regenotyping exercise (involving 118 samples for 7 loci), only five genotyping errors were found; a genotyping error rate of 0.3%. Three of these errors were allelic dropouts, the other two either false alleles or contaminations. The error rate in this study was low (see Bonin et al. 2004) and therefore unlikely to have notably influenced results from any analysis of the dataset.

### Descriptive genetics

No statistically significant differences in genetic composition between the two cohorts sampled in 2006 and 2007 were observed in any of the sampling locations or between the temporally spaced Faughan and Deele samples. Thus, all temporal samples representing all 13 sampling locations were pooled prior to subsequent analyses.

The mean number of alleles per locus varied between 12.9 in sample Owenreagh East and 18.4 in sample Faughan. The average expected heterozygosity (Table 3) was very similar over all samples and ranged between 0.84 (Owenreagh South) and 0.88 (Fairy Water). Average allelic richness over loci (Table 3) varied from 11.99 (Owenreagh South) to 14.83 (Faughan). While a few instances of significant (α = 0.05) genotypic linkage disequilibrium were detected in a few samples, none of these were found to be relevant after Bonferroni correction for multiple tests. Similarly, only very few cases involving deviation from HWE were observed; and these involved not more than two loci per sample. None of these were found to remain significant following Bonferroni correction.

**Table 3:** Expected (*H*_E_) and observed heterozygosity (*H*_O_), number of alleles per locus (*N*_A_), allelic richness (*A*_R_), and inbreeding coefficient (*F*_IS_). Samples showing significant deviation from Hardy–Weinberg equilibrium (HWE) are denoted with an asterisk (*)

Locus	Fau	Gle	Den	Owe	Ows	Dru	Fin	Ree	Der	Str	Cam	Fai	Dee	Mean
Sssp1605														
*H*_o_	0.73	0.76	0.68	0.77	0.74	0.84	0.78	0.82	0.73	0.88	0.69	0.85	0.77	0.77
*H*_E_	0.77	0.81	0.66	0.77	0.76	0.79	0.78	0.74	0.79	0.80	0.77	0.83	0.74	0.77
*N*_a_	9	9	7	8	9	11	10	12	10	7	6	9	8	8.8
*A*_r_	7.36	8.44	6.25	8	8.07	9.16	9.11	9.62	8.72	7	6	9	7.58	8.02
*F*_is_	0.04	0.06	–0.02	0.01	0.03	–0.07	0.00	–0.12	0.07	–0.10	0.11	–0.03	–0.03	
HWE	–	–	–	–	–	–	–	–	–	–	–	–	–	
Sssp2201														
*H*_o_	0.91	0.86	0.95	0.92	0.92	0.91	0.93	0.97	0.94	0.90	0.94	0.81	0.93	0.91
*H*_E_	0.94	0.93	0.94	0.92	0.92	0.93	0.94	0.91	0.93	0.95	0.91	0.93	0.94	0.93
*N*_a_	30	21	25	18	18	23	26	23	25	22	23	20	28	23.2
*A*_r_	24.4	19.2	23.3	18	17	19.5	22.6	19.6	22.6	22	23	20	24.8	21.23
*F*_is_	0.03	0.07	–0.01	0.01	0.00	0.02	0.01	–0.06	0.00	0.06	–0.03	0.13	0.03	
HWE	–	*	–	–	–	–	–	–	–	*	–	*	–	
Sssp2210														
*H*_o_	0.72	0.85	0.76	0.79	0.69	0.76	0.71	0.63	0.79	0.81	0.83	0.81	0.85	0.77
*H*_E_	0.79	0.82	0.79	0.83	0.77	0.75	0.73	0.71	0.76	0.79	0.79	0.80	0.81	0.78
*N*_A_	10	12	9	10	10	9	12	10	12	10	7	11	11	10.2
*A*_r_	9.6	11.1	8.72	10	9.09	8.93	10.3	9.7	10.2	10	7	11	10.8	9.73
*F*_is_	0.09	–0.05	0.04	0.04	0.11	–0.01	0.03	0.12	–0.04	–0.03	–0.06	–0.01	–0.05	
HWE	–	–	–	–	–	–	–	–	–	–	–	–	–	
Sssp2216														
*H*_o_	0.84	0.79	0.93	0.90	0.79	0.80	0.91	0.82	0.93	0.94	0.92	0.83	0.90	0.87
*H*_E_	0.91	0.85	0.90	0.84	0.81	0.87	0.91	0.91	0.89	0.90	0.87	0.91	0.89	0.88
*N*_A_	20	15	15	11	12	11	17	16	16	14	12	12	13	14.2
*A*_r_	16.5	12.7	14	11	11.1	10.4	14.7	14.2	14.8	14	12	12	12.2	13.05
*F*_is_	0.07	0.07	–0.03	–0.06	0.03	0.08	0.01	0.10	–0.04	–0.04	–0.05	0.08	–0.01	
HWE	–	–	–	–	–	–	–	–	–	–	–	–	–	
SsspG7														
*H*_o_	0.84	0.93	0.88	0.90	0.84	0.83	0.88	0.89	0.92	0.92	0.94	0.85	0.89	0.88
*H*_E_	0.88	0.89	0.86	0.86	0.87	0.87	0.91	0.89	0.89	0.88	0.86	0.86	0.85	0.88
*N*_A_	18	16	14	15	14	14	13	18	16	14	14	14	14	15.3
*A*_R_	13.7	14.6	12.3	15	12.5	12.3	17.1	16	15.1	14	14	14	13.2	14.15
*F*_1S_	0.05	–0.04	–0.02	–0.05	0.03	0.04	0.04	0.00	–0.03	–0.04	–0.09	0.01	–0.04	
HWE	–	–	–	–	–	–	–	–	–	–	–	–	–	
Ssa197														
*H*_o_	0.91	0.90	0.93	0.88	0.89	0.92	0.94	0.78	0.95	0.96	0.92	0.94	0.91	0.91
*H*_E_	0.91	0.90	0.90	0.90	0.91	0.92	0.91	0.90	0.92	0.93	0.93	0.92	0.91	0.91
*N*_A_	28	20	20	17	17	24	20	20	19	23	21	IS	19	20.5
*A*_R_	20.1	17.4	17.1	17	16	20.7	17.2	16.8	17.1	23	21	IS	16.2	18.27
*F*_is_	0.01	0.01	–0.03	0.03	0.03	0.01	–0.03	0.13	–0.03	–0.04	0.02	–0.02	0.00	
HWE	–	*	–	–	–	–	–	*	–	–	–	–	–	
Ssa202														
*H*_o_	0.79	0.94	0.89	0.83	0.86	0.83	0.90	0.86	0.90	0.81	0.88	0.90	0.93	0.87
*H*_E_	0.89	0.89	0.87	0.87	0.86	0.83	0.87	0.88	0.87	0.86	0.86	0.89	0.88	0.87
*N*_A_	14	12	12	11	11	13	13	12	11	11	11	12	11	11.8
*A*_R_	12.2	11.4	11.6	11	10.2	11.6	12.3	11.8	10.7	11	11	12	10.2	11.30
*F*_is_	0.11	–0.06	–0.01	0.04	–0.01	–0.01	–0.03	0.02	–0.03	0.06	–0.02	–0.01	–0.05	
HWE	*	–	–	–	–	–	–	–	–	–	–	–	–	
*N*	96	96	96	48	96	96	96	96	96	48	48	48	48	
Mean														
*H*_o_	0.82	0.86	0.86	0.85	0.82	0.84	0.86	0.82	0.88	0.89	0.87	0.86	0.88	
*H*_E_	0.87	0.87	0.85	0.86	0.84	0.85	0.87	0.85	0.87	0.87	0.86	0.88	0.86	
*N*_A_	18.4	15.0	14.6	12.9	13.0	15.0	16.6	16.8	15.6	14.4	13.4	13.7	14.9	
*A*_R_	14.83	13.54	13.31	12.86	11.99	13.22	14.76	13.97	14.19	14.43	13.43	13.71	13.56	
*F*_is_	0.06	0.01	–0.01	0	0.03	0.01	0.01	0.03	–0.01	–0.02	–0.02	0.02	–0.02	

Genetic differentiation, estimated as pairwise *F*_ST_ and *D*_est_ between sampling locations, ranged from 0.001 to 0.06 for *F*_ST_ and from 0.003 to 0.3 for *D*_est_ (Table 4). In general, *D*_est_ estimates were found to be in the order of three to four times higher than their *F*_ST_ equivalents. All pairwise comparisons were found to be statistically significant with one exception involving samples from the Finn and Reelan. Global *F*_ST_ estimate was 0.02, while the global *D*_est_ estimate (calculated as an approximation of the harmonic mean of *D*_est_ values over all loci using the following equation: Hmean ∼ 1/ [(1/A) + var(D)(1/A)^3^] where A is the arithmetic mean and var(D) is the variance of D_est_ values over loci) was 0.12 (i.e., sixfold higher). Despite ongoing debate over the application Jost's *D*, and the merits of using *F*_ST_ for microsatellite data, we found a near-perfect correlation between both estimates (*r* = 0.96, *P*≤ 0.001) in the present dataset. Interesting, while we also found a strong positive correlation between *D*_C_ and *D*_est_ (*r* = 0.81, *P*≤ 0.0001), this was not as strong as between *F*_ST_ and *D*_est_.

**Table 4:** Matrix showing pairwise *F*_ST_ values, *D*_est_ (in Italics) (above diagonal), and geographical distance between sampling locations (below diagonal) in the Foyle River in kilometers.Non significant *D*_est_ and *F*_ST_ values after sequential Bonferroni correction are given in bold

	Fau	Gle	Den	Owe	Ows	Dru	Fin	Ree	Der	Str	Cam	Fai	Dee
Fau	–	0.014/*0.047*	0.009/*0.021*	0.010/*0.023*	0.034/*0.211*	0.01*8/0.117*	0.011/*0.066*	0.018/*0.096*	0.010/*0.059*	0.005/*0.043*	0.011/*0.046*	0.008/*0.033*	0.007/*0.053*
Gle	95.71	–	0.023/*0.112*	0.017/*0.069*	*0.025/0.111*	*0.025/0.121*	0.015/*0.077*	0.021/*0.127*	0.016/*0.070*	0.012/*0.045*	0.014/*0.065*	0.012/*0.054*	0.014/*0.068*
Den	49.76	54.56	-	0.017/*0.068*	*0.055/0.301*	*0.036/0.217*	*0.022/0.129*	0.029/*0.156*	0.022/*0.119*	0.019/*0.084*	0.017/*0.074*	0.017/*0.085*	0.009/*0.044*
Owe	106.34	29.56	66.82	–	0.031/*0.139*	*0.025/0.127*	*0.025/0.158*	0.033/*0.190*	0.021/*0.113*	0.015/*0.066*	0.015/*0.065*	0.016/*0.086*	0.014/*0.052*
Ows	132.36	67	94.19	76.37	-	*0.026/0.113*	*0.036/0.161*	0.041/*0.194*	0.036/*0.150*	0.030/*0.160*	0.031/*0.167*	0.034/*0.196*	0.042/*0.208*
Dru	125.57	63.32	88.16	71.98	37.18	-	*0.026/0.113*	0.031/*0.133*	0.025/*0.109*	0.021/*0.077*	0.020/*0.096*	0.015/*0.095*	0.028/*0.181*
Fin	75.78	68.39	37.96	79.89	102.64	98.76	-	**0.001**/*0.003*	0.002/*0.009*	0.011/*0.068*	0.019/*0.119*	0.010*/0.045*	0.015/*0.092*
Ree	99.52	92.13	61.7	103.85	126.38	122.5	24.14	–	0.009/*0.035*	0.021/*0.101*	0.022/*0.117*	0.015/*0.068*	0.022/*0.124*
Der	98.67	59.09	59.82	72.77	95.34	90.9	72.19	95.93	–	0.007/*0.029*	0.018/*0.090*	0.008/*0.038*	0.015/*0.115*
Str	89.9	27.92	53.26	36.43	44.9	42.62	62.55	86.29	53.09	–	0.010/*0.043*	0.008/*0.044*	0.010/*0.066*
Cam	119.68	57.75	83.11	66.23	47.39	44.24	92.43	116.17	85.15	34.67	–	0.007/*0.027*	0.013/*0.099*
Fai	102.96	38.55	63.14	49.44	39.34	35.12	75.63	99.37	62.18	17.8	29.17	–	0.013/*0.105*
Dee	65.45	65.15	30.31	77.48	102.36	97.97	39.03	72.28	66.62	92.26	92.26	72.98	–

### Geographical population structure

**Figure 2:**
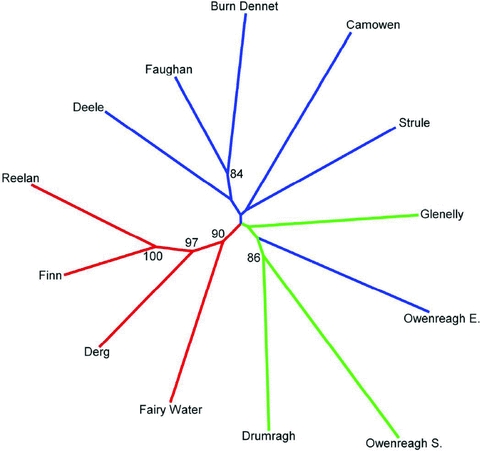
Unrooted Neighbor-joined phylogram based on Cavalli-Sforza and Edwards’ chord (*D*_C_) genetic distance. Bootstrap values in percentage of 10,000 replicates. Only bootstrap values over 60 are shown. Colors of branches indicate group membership as indicated by STRUCTURE analysis; Red = West, Blue = Northeast, and Green = Southeast.

The NJ tree based on the Chord (*D*_C_) genetic distance measure (Fig. 2) confirmed the existence of several population groupings within the Foyle catchment, which were supported by moderate/high bootstrap support. These groups generally adhere to the geographical origin of particular samples within the River Foyle system, thus samples from the western, northeastern, and southeastern tributaries fall into three reasonably well-defined clusters. One of these geographical groups (western), identified by both NJ and STRUCTURE analyses, consists of geographically proximate samples from the western of the catchment. While the other two groups are characterized by geographically close samples (e.g., Faughan and Burn Dennet in the north and the Owenreagh South and Drumragh in the south) from the extreme ends of the catchment (i.e., south and north of the catchment), the analyses include other samples that are geographically inconsistent with the groups (e.g., eastern samples Camowen and Strule grouping with the north and the eastern samples of Glenelly and Owenreagh East grouping with the south).

There was significant overall positive association between geographical distance and *F*_ST_ genetic distance among the samples shown by the Mantel test (*r* = 0.293, *P* = 0.039; Table 5A). Not surprising, a similar positive association was also observed with *D*_est_ (*r* = 0.332, *P* = 0.009). This observed overall positive IBD pattern, however, was strongly influenced by the major regions (i.e., western, northeastern, and southeastern). Within each individual region, there was no evidence of IBD, with the exception of the western regional group. In the latter case, however, given small number of samples within the group, results should be interpreted with caution. To test the independent effects of both geographical distances and the major geographical regions, as identified by the previous analyses (e.g., STRUCTURE), in the patterns of population differentiation observed, partial Mantel tests were carried out (Table 5B). Genetic distances (*F*_ST_ and *D*_est_) were found to be significantly correlated to both geographical distances and regions, with the latter variable having a more significant effect. Together, these analyses provide unambiguous evidence for IBD in the River Foyle among regions, but not within all regions per se.

**Table 5:** (A) Mantel test results for correlation between genetic (FST and Dest) and geographic distances for both the Foyle system and for the major geographical groups identified during analysis; (B) Partial Mantel test results to investigate the independent regional and geographic effects on the genetic distance (FST and Dest). P-values derived from 10,000 iterations. Significant results (P < 0.05) given in bold

Group	*F*_ST_		*D*_est_	
		
	*r*-value	*P*-value	*r*-value	*P*-value
**(A)**				
All 13 2006–2007 sampling sites	0.293	**0.039**	0.332	0.009
Northeast sampling sites only	–0.142	0.692	–0.193	0.757
Southeast sampling sites only	–0.790	0.828	0.237	0.658
West sampling sites only	0.729	**<0.001**	0.850	**<0.001**
**(B)**				
Partial Mantel test				
Genetic and geographical distance controlling for regional effects	0.336	**0.033**	0.340	**0.023**
Genetic distance and region controlling for geographical distance effects	0.431	**<0.001**	0.470	**<0.001**

**Figure 3:**
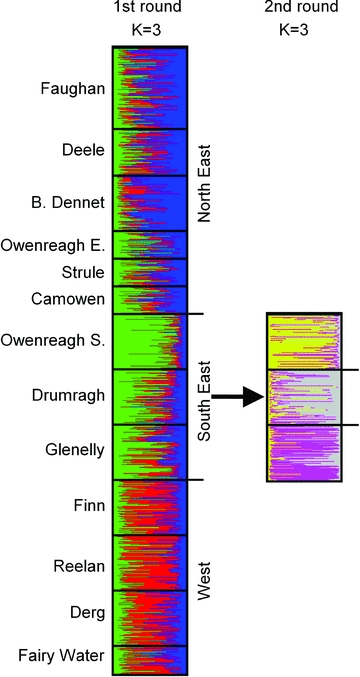
STRUCTURE bar plots (aligned using CLUMPP) depicting population structure based on two hierarchical rounds of analysis; *K* = 3 (first round, left) and *K* = 3 (second round, right) where membership of each individual to the different clusters can be deducted from the proportion of each color in the bars.

The results of the STRUCTURE analysis are in agreement with the Chord genetic distance clustering in supporting three major genetic groups within the Foyle catchment, which are generally defined by geography (i.e., western, northeastern, and southeastern tributaries). Interestingly, at the most basic level of structure, Δ*K* was found to be bimodal at *K* = 3 and *K* = 4. Close examination of the graphical output of the STRUCTURE analyses (Fig. 3) suggests some evidence for further partitioning within the northeastern group. Thus, the eastern samples from the Owenreagh East, Camowen, and Strule appear to be somewhat genetically distinct from samples from the Faughan, Deele, and the Dennet. Additional STRUCTURE hierarchical analyses, however, failed to indentify additional structuring within this group. This was not the case for the southeastern cluster in which further substructuring was uncovered from the hierarchical STRUCTURE approach. Thus, the Owenreagh, the Drumragh, and the Glenelly are clearly genetically distinct units (Fig. 3). The results from STRUCTURE and the *D*_C_ clustering were also confirmed by the Barrier analysis in separating the three major regional groupings within the Foyle System and also, to a large extent, the substructuring within the groups (results not shown).

### Geographical partition of genetic variance

The results of the AMOVA analysis, carried taking in consideration (1) the main regions within the Foyle System (i.e., west, northeast, and southeast); (2) samples within regions; and (3) samples within the Foyle System, are summarized in Table 6. While 97.7% of the observed genetic variation is contained within samples, a significant (*P* < 0.001) percentage of the observed genetic variance is explained by differences among samples within (1.35%), and among regions (0.98%), thus confirming previous analyses.

**Table 6:** AMOVA among the Atlantic salmon samples from the River Foyle system

Variance component	df	Percentage of total variance	*P*-value
Among regions	2	0.98	<0.001
Among samples within regions	12	1.35	<0.001
Within samples	2157	97.7	<0.001

## Discussion

The overall levels of genetic variability in salmon of the Foyle River system (measured as heterozygosity, allelic richness, and mean number of alleles per locus) reported in this study are at the high end of the range reported for similar population genetic studies using microsatellite markers on Atlantic salmon (e.g., Primmer et al. 2006; Dillane et al. 2007; Garant et al. 2000). This is possibly associated with the very large Atlantic salmon stock within the Foyle River system.

Similar to previous studies examining within river system population structuring (e.g., Dillane et al. 2008), substantial levels of genetic differentiation were observed among the River Foyle tributary samples. Given lack of differentiation between the temporal samples examined in this study, it is reasonable to assume that the patterns of population genetic structuring observed within the Foyle River system are temporally stable. It is worth noting that one of the temporal samples was collected from a relatively small tributary in which the resident salmon population is believed to be small (i.e., average of 62 annual spawners over a 10-year period, Loughs Agency Annual Report 2005).

The higher levels of genetic differentiation revealed by *D*_est_ relative to *F*_ST_ are in line with Jost (2008), who argued that *F*_ST_ (*G*_ST_) tends to underestimate true levels of population differentiation compared to Jost's *D* and related statistics (e.g., Hedrick 2005). Both methods, however, were in agreement with respect to the patterns of population structure observed within the Foyle system. The differences in the magnitude of the observed differences, however, could have implications from a management viewpoint if conservation of population structure and genetic differentiation are the focus. For instance, on the basis of low *F*_ST_ estimates, populations that would look only marginally differentiated and perhaps not worthy of discrete population status and possibly associated specific conservation measures, would have to be classified as highly differentiated on the basis of *D*_est_ and therefore might justify a higher level of protection.

It is important to note that, in many ways, this discussion arises because of a misunderstanding of *F*_ST_ statistics as it relates to microsatellite data, as opposed to low or moderately polymorphic allozyme data for which this statistic was particularly suited for. As clearly demonstrated by Hedrick (2005) and Jost (2008), standard *F*_ST_ estimates from highly polymorphic microsatellite data are often biased toward the lower end of the differentiation scale (i.e., 0 = nondifferentiated; to 1 = completely differentiated populations). Thus, there is an inherent methodological penalty associated with the use of this class of markers with this statistic. Many authors, however, still fail to acknowledge or recognize this important issue, with the unfortunate consequence that significant genetic differences are often dismissed as being inconsequential on the basis of “small”*F*_ST_ estimates. Both Hedrick (2005) and Jost (2008) have suggested potential adjustments to this statistic to account for the bias. This, however, has not as yet been generally implemented in studies that have been reported in the literature. A potential reason for this is the fact that little is still known about the behavior of these “adjusted”*F*_ST_ estimates in relation to the evolutionary factors they are measuring, for example, gene flow, mutation, random genetic drift, and selection (but see Whitlock 2011). Thus, while traditional *F*_ST_ statistics can be readily interpreted in terms of the above, this is not necessarily the case for the “adjusted”*F*_ST_ statistics. Recently, Whitlock (2011) suggested that the traditional *F*_ST_ statistics perform better than the “adjusted” equivalent measures. In here, however, based upon evaluation of empirical data, we found no evidence supporting this argument beyond the reported differences in magnitude. Thus, further studies are still urgently required to address this important issue.

In instances where these “adjusted” measures of genetic differentiation have been implemented, results have been inconclusive. For instance, Hoffman et al. (2009) did not find substantial evidence to support the better performance of Jost's *D*_est_ as compared to standard *F*_ST_ in detecting differentiation in Steller's sea lion (*Eumetopias jubatus*). We argue, however, that levels of microsatellite polymorphisms observed in the Steller's sea lion (4–11 alleles over 13 loci) are comparatively low in relation to what is observed in other species (e.g., 13–37 alleles over seven loci in the present study for Atlantic salmon). Thus, the bias associated with high levels of genetic diversity as discussed by Hedrick (2005) and Jost (2008) was potentially less evident in Hoffman et al's. (2009) study in comparison to what was observed for Atlantic salmon in River Foyle.

As conservation and management strategies of many salmonid fish species have been predominantly based on population differentiation estimates of *F*_ST_, as recommended by Heller and Siegismund (2009), we argue that the use of either Hedrick's (2005) standardized genetic statistics or Jost's (2008)*D*_est_ should provide a more appropriate measurement of population differentiation. Indeed, we suggest that reviews should be carried out to reassess the levels and patterns of population divergence from studies solely relying on uncorrected *F*_ST_. This should be a priority for valuable Atlantic salmon populations where the ability to accurately identify individual population units is critical. Furthermore, from a conservation perspective, it would be relevant to inform managers about the caveats of the interpretation of standard *F*_ST_ previously estimated and presented insofar as microsatellite data are concerned.

Notwithstanding the issue discussed above, for a comparative perspective between this and other genetic studies on Irish Atlantic salmon populations using similar microsatellite markers, it is worth noting that the global uncorrected *F*_ST_ values (*F*_ST_ = 0.02) did not differ greatly. For instance, Dillane et al. (2008) reported global *F*_ST_ values of 0.024 for the Moy River in the west of Ireland. *D*_est_ estimates, in the Foyle, both over all loci and population samples were on average three to four times higher compared to global *F*_ST_ values. Again, using the argument above, if this study had been limited to only using *F*_ST_ as a measure of genetic differentiation, the vast majority of differentiation between samples could have been perceived as very small. Given the argument above, the important point is whether the statistic is significant or not, while the value itself is largely irrelevant.

Given observed differences in allelic frequency distribution (exact tests), the majority of the samples examined from the Foyle represent more or less independent temporally stable population units (i.e., with limited levels of gene flow). The only exception (i.e., no genetic differences) involved the geographically close sampling locations Finn and Reelan (western region, Fig. 1). Given that these samples were taken from within the same tributary river, the lack of genetic differentiation between them is not entirely surprising.

Despite genetic differences observed among individual samples, our data suggest the existence of three major population groupings (West, Southeast, and Northeast) in the Foyle catchment, the overall pattern of which generally accords with the geographical and hydrological organization of the catchment, and which is consistent with a member-vagrant evolutionary model. However, while the membership of samples belonging to the West group is geographically well defined, some inconsistencies were observed in the membership of the Northeastern and Southeastern groups. We argue below that these discrepancies can be explained by anthropogenically mediated disturbances.

The western tributaries comprise the largest rivers in the Foyle catchment with correspondingly large population sizes. In comparison to the other areas of the catchment, the western rivers are considered to be reasonably pristine from an environmental perspective. This area of the catchment is also well known for the presence of multi-sea winter fish, a life-history trait that is uncommon in the rest of the catchment. Thus, the genetic uniqueness of samples from this area is likely to reflect an independent evolutionary history. Overall, there was no strong evidence for IBD within groups, which suggests high levels of conductivity and therefore is in agreement with the concept of a metapopulation evolutionary model.

Within the Northeast group, the most northern tributaries (i.e., Faughan, Deele, and Burn Dennet), similar to those in the west of the catchment, are also large, with good salmon habitat and supporting large populations. Indeed, available demographic data for the Foyle system (authors’ unpublished data) suggest that the tributaries from the north and from the west of the catchment are responsible for the most of the current salmon productivity in the Foyle (i.e., ∼80%). In contrast, however, the population sizes of the eastern (Glenelly, Owenreagh East, Camowen, and the Strule) and southeastern (Owenreagh South and Drumragh) tributaries, despite their large habitat area (between 25% and 35% of the catchment) are relatively narrow and small (authors’ unpublished data). This part of the Foyle catchment is characterized by intensive farming and it is heavily impacted by arterial drainage schemes resulting in the disturbance of natural habitats, which severely limit productivity. Therefore, the genetic pattern characteristic of samples belonging to this region reflects random stochastic processes (e.g., genetic drift acting on small gene pools), associated with anthropogenetic impacts, such a loss of spawning habitats or poor water quality, as the main factors shaping contemporary population genetic structure in these regional groupings.

This hypothesis is supported by the lack of evidence for substructuring as provided by both NJ and the STRUCTURE analyses. Alternatively, the observed similar genetic makeup of samples from both regions could reflect past historical gene flow prior to the development of the extensive arterial drainage scheme in the eastern and southern regions of the catchment. At the moment, the data are insufficient to disentangle both hypotheses.

While we observed strong and statistically significant positive correlation between geographical distance and genetic distance (IBD) within the River Foyle Atlantic salmon stock, this trend was linked to the three indentified genetic regional groupings rather than individual samples. Interestingly, IBD within a single river system is relatively uncommon in Atlantic salmon. Evidence for IBD is more frequently reported from studies on Atlantic salmon population genetic structure at larger spatial scales often involving multiple river catchments (e.g., McConnell et al. 1997; King et al. 2001; and Dillane et al. 2007).

The Atlantic salmon stock of the River Foyle most likely exists as several distinct, long-term temporally stable, populations within well-defined geographical regions, in which migration occurs more frequently than between populations from different regions. It is clear from our findings that the genetic structure of the River Foyle Atlantic salmon stock is complex. Thus, it cannot be simply defined within a single evolutionary model. If considering the whole system, the population structure of salmon within the Foyle catchment is better explained under the member-vagrant evolutionary model as confirmed by the clear presence of IBD at this level. However, within each of the major genetic regions identified, the metapopulation model best explains the observed structuring, as there is no evidence for IBD at this level. We argue that these models are not mutually exclusive and can operate simultaneously on different geographical scales within a single river system. Thus, any attempts to identify a best model fit would be artificial and not reflective of the complex evolutionary history commonly associated with the species.

Interestingly, Garant et al. (2000) has previously argued that the member-vagrant evolutionary model was too rigid to explain the population genetic structure of Sainte-Marguerite River salmon populations. In their study, the authors found difficult to accommodate extinction–colonization events, lack of temporal genetic stability, and absence of an IBD with a pure member-vagrant hypothesis, as these processes are more associated with the metapopulation model. Similarly, while Primmer et al. (2006) concluded that the member-vagrant evolutionary model best explained the genetic structure observed in the Varzuga River, they have also reported possible extinction–colonization events prompting a consideration of metapopulation processes acting at some sites.

From a conservation and management viewpoint, focus should be orientated toward identifying and preserving appropriate evolutionary entities. In the Foyle, using genetic evidence and an environment quality indicator (EQI), it is evident that conservation efforts should be targeted toward southern and eastern tributary catchments. This could be done by restoring the integrity of habitats prior to the development of intensive farming activities and associated drainage schemes. The data also suggest that the management of salmon in the Foyle should accommodate the three regional groupings identified in this study. Future studies should continue to explore the interplay between the evolutionary models determining genetic structure and to what extent the findings reported in here apply elsewhere.

## Data Archiving

Genotypes and all other raw data used in this study are stored on an electronic database at AFBINI. At request available to others via the corresponding author.
